# A survey of surgical patients’ perspectives and preferences towards general anesthesia techniques and shared-decision making

**DOI:** 10.1186/s12871-023-02219-5

**Published:** 2023-08-17

**Authors:** Bethany R. Tellor Pennington, Mary C Politi, Arbi Ben Abdallah, Allison M. Janda, Ingrid Eshun-Wilsonova, Nastassjia G. deBourbon, Lilly Siderowf, Heidi Klosterman, Sachin Kheterpal, Michael S. Avidan

**Affiliations:** 1 Department of Anesthesiology, Washington University School of Medicine, St. Louis, MO USA; 2 Department of Surgery, Division of Public Health Sciences, Washington University School of Medicine, St. Louis, MO USA; 3 Department of Anesthesiology, University of Michigan, Ann Arbor, MI USA; 4 Department of Medicine, Washington University School of Medicine, St. Louis, MO USA; 5 Department of Emergency Medicine, Magnolia Regional Health Center, Corinth, MS USA; 6 College of Arts and Sciences, Washington University, St. Louis, MO USA; 7 Patient Partner, St. Louis Missouri, USA

**Keywords:** Total intravenous anesthesia, Inhaled volatile anesthesia, Patient engagement, Patient preference

## Abstract

**Background:**

The decision about which type of general anesthetic to administer is typically made by the clinical team without patient engagement. This study examined patients’ preferences, experiences, attitudes, beliefs, perceptions, and perceived social norms about anesthesia and about engaging in the decision regarding general anesthetic choice with their clinician.

**Methods:**

We conducted a survey in the United States, sent to a panel of surgical patients through Qualtrics (Qualtrics, Provo, UT) from March 2022 through May 2022. Questions were developed based on the Theory of Planned Behavior and validated measures were used when available. A patient partner who had experienced both intravenous and inhaled anesthesia contributed to the development and refinement of the questions.

**Results:**

A total of 806 patients who received general anesthesia for an elective procedure in the last five years completed the survey. 43% of respondents preferred a patient-led decision making role and 28% preferred to share decision making with their clinical team, yet only 7.8% reported being engaged in full shared decision making about the anesthesia they received. Intraoperative awareness, pain, nausea, vomiting and quickly returning to work and usual household activities were important to respondents. Waking up in the middle of surgery was the most commonly reported concern, despite this experience being reported only 8% of the time. Most patients (65%) who searched for information about general anesthesia noted that it took a lot of effort to find the information, and 53% agreed to feeling frustrated during the search.

**Conclusions:**

Most patients prefer a patient-led or shared decision making process when it comes to their anesthetic care and want to be engaged in the decision. However, only a small percentage of patients reported being fully engaged in the decision. Further studies should inform future shared decision-making tools, informed consent materials, educational materials and framing of anesthetic choices for patients so that they are able to make a choice regarding the anesthetic they receive.

**Supplementary Information:**

The online version contains supplementary material available at 10.1186/s12871-023-02219-5.

## Background

For surgical procedures that require general anesthesia, there are several effective anesthetic options including inhaled volatile agents and intravenous agents such as propofol. However, the decision about which type of anesthesia to administer is typically made by the clinical team without patient engagement. While some patient factors or surgical procedures require the selection of one option over the other, there are many patients and types of surgical procedures for which either could be safely administered. Clinician-led decision making in this context is likely influenced by a combination of clinician beliefs and preferences, and lack of patient recognition about options [1–4]. In addition, clinicians may not have compelling evidence to support a detailed discussion of trade-offs between anesthetic agents. In situations of uncertainty about the best option from a medical standpoint, patients’ preferences are essential to consider to support high-quality, patient-centered care plans [5–7].

Studies about patient preferences and concerns about anesthesia are sparse [8–12], but have shown that patients are more fearful and anxious about general anesthesia compared to local or regional anesthesia [11–13]. Patients have expressed concerns about postoperative pain, waking up during surgery, not waking up after surgery, permanent disability, and postoperative nausea and vomiting [8, 9]. The recent National Poll on Healthy Aging survey reported over 50% of older adults who considered elective surgery were concerned about pain or discomfort and difficulties with recovery [14]. It remains unknown whether or not a specific general anesthetic technique, total intravenous or inhaled volatile anesthesia, influences the incidence of these common patient concerns, yet, some studies suggest that patients may actually prefer inhaled anesthesia over intravenous [12]. If one method of general anesthesia administration were superior in relation to one or more of these patient concerns, conversations about trade-offs between options would be essential prior to surgery. These conversations could support patient-centered decision making and adequate informed consent about anesthesia.

The Trajectories of Recovery after Intravenous Propofol vs. inhaled VolatilE anesthesia (THRIVE) trial (NCT05346588 [15],) aims to explore which type of anesthesia [Propofol total intravenous anesthesia (TIVA) or inhaled volatile anesthesia] results in a better patient experience. Understanding which anesthetic outcomes are most important to patients will ensure patient preferences and perceptions are prioritized in the THRIVE trial. In addition, this information can inform future shared decision-making tools and framing of anesthetic choices for patients.

To complement the THRIVE trial evaluating the clinical and patient-centered differences in experiences with total intravenous and inhaled volatile anesthesia, we conducted a survey among patients who had elective surgery with general anesthesia within the past five years. We explored patients’ preferences, experiences, attitudes, beliefs, perceptions, and perceived social norms about anesthesia and about engaging in the decision regarding anesthetic choice with their clinician.

## Methods

We developed a survey based on the Theory of Planned Behavior [16–18]. This theory states that attitudes, subjective norms, perceived control and intention can influence behavior, such as the selection of an anesthesia type. We used validated outcome measures when available in order to explore patients’ attitudes, beliefs, perceived social norms, and self-efficacy about anesthesia choices and about shared decision-making. Questions were developed based on this theory and from additional related studies assessing these constructs [19, 20]. A patient partner who had experienced both intravenous and inhaled anesthesia contributed to the development and refinement of the questions. This study was deemed exempt by the Institutional Review Board (IRB) of Washington University School of Medicine in St. Louis, Missouri (IRB 202,203,072).

### Participants and procedures

This survey was sent to a panel of surgical patients through Qualtrics panels (Qualtrics, Provo, UT) from March 2022 through May 2022. Qualtrics panels can provide convenience samples that represent various demographic groups in the U.S [21]. Participants were considered eligible if they were United States residents 18 years of age or older who received general anesthesia for elective surgery in the last five years. If they met eligibility criteria and agreed to continue, the respondents were then asked to complete the 38-question survey (See Appendix 1). Participants who met eligibility criteria and started the survey but either completed the survey too quickly (in less than 3 min), started the survey after the planned enrollment number was reached, or did not complete enough questions were automatically excluded according to the pre-specified criteria determined with Qualtrics.

The study was hypothesis-generating rather than hypothesis-testing. We calculated the sample we would need to explore our outcomes based on the smallest sample estimated that would reflect the views of the general population who experienced elective surgery. With an annual population size of surgical patients varying from five to seven million a year, we assumed an incident rate of elective surgical procedures to be 10–20% over five years in adult patients. The required sample needed was 664 to provide a confidence level of 95% or 99% and an acceptable margin of sampling error varying between 1% and 5% (the smaller the margin of sampling error, the higher the confidence in the data provided by the survey). With an estimated 20% margin of incomplete surveys, we anticipated needing a sample of roughly 800 completed surveys.

### Measures

#### Attitudes

Preferences for anesthesia, concerns and important factors that can influence type of anesthesia, were assessed using Likert Scale questions. The control preferences scale [22] was used to measure patients’ preferences for involvement in decision making, ranging from patient-directed to share to clinician-directed decisions. Previous surgical feelings about satisfaction and worries were derived from the National Cancer Institute Health Information National Trends 5-Cycle 4 Survey questions [23].

Additional items assessed included questions regarding previous anesthesia experiences because prior experiences with similar situations can influence preferences [24]. The 11-item Trust in Physician Scale was used to measure medical trust/mistrust, scored on a 5-point Likert scale ranging from 1 (strongly disagree) to 5 (strongly agree). A summary measure of trust is obtained by taking the unweighted mean of the responses to the 11 questions and transforming that value to a 0–100 scale. Higher scores reflect greater trust; mean trust scores in general range from about 45 (lower end of trust) to 83 (higher end of trust) [25, 26].

#### Beliefs

Questions pertaining to beliefs about anesthesia were derived from the National Cancer Institute Health Information National Trends 5-Cycle 4 Survey questions [23].

#### Perceived social norms and shared decision making

The collaboRATE measure [27, 28] was used to assess the degree of shared decision making that occurred. We analyzed this using the “top score” method as recommended by the scoring guidelines. Respondents were also asked what they believed other surgical patients would think about engaging in the shared decision making process. Additional questions assessed whether or not the anesthesia clinician encouraged them to ask questions and if the anesthesia clinician made them feel relaxed.

#### Self-efficacy

The decision self-efficacy scale [29] was used to assess confidence in making a choice, using 6 of the original 11 questions that were relevant to this study. Additional questions pertaining to confidence and seeking information were derived from the National Cancer Institute Health Information National Trends 5-Cycle 4 Survey questions [23].

#### Sociodemographic information

Demographic characteristics including age, gender, race, education, insurance status, and area of residence were also assessed.

### Data analysis

All survey data were entered into a database and processed using SAS(R) Proprietary Software 9.4 for WINDOWS (SAS Institute Inc., Cary, NC). The statistical analysis consisted of a descriptive as well as a bivariate analysis. Descriptive statistics (frequencies and percentages for categorical variables and mean (SD) for continuous variables and Likert scales) were performed on all survey variables. Bivariate analyses were conducted to determine the association of specific respondents’ variables with their socio demographic grouping, including age, gender and race. We explored how these attributes vary by age and gender as past literature suggests that these demographic characteristics may influence anesthetic pharmacokinetics and subsequent patient experiences after receiving general anesthesia [30–34]. Differences by race were explored as racial disparities may exist pertaining to perioperative complications and recovery experiences in surgical patients [35–37]. Any differences found were reported in the results section. Differences between two groups were tested using Chi-square statistics for binary variables or unpaired Student t-test for quantitative variables, appropriately. More than two groups were assessed for differences with analysis of variance (ANOVA) using Tukey method for post hoc analysis. The p-values from these statistical procedures were adjusted using Bonferroni correction method to adjust for multiple comparisons. All statistical testing was two-sided and a p-value < 0.05 was regarded as statistically significant. Partially completed questions were not included in these analyses.

## Results

**Fig. 1:**
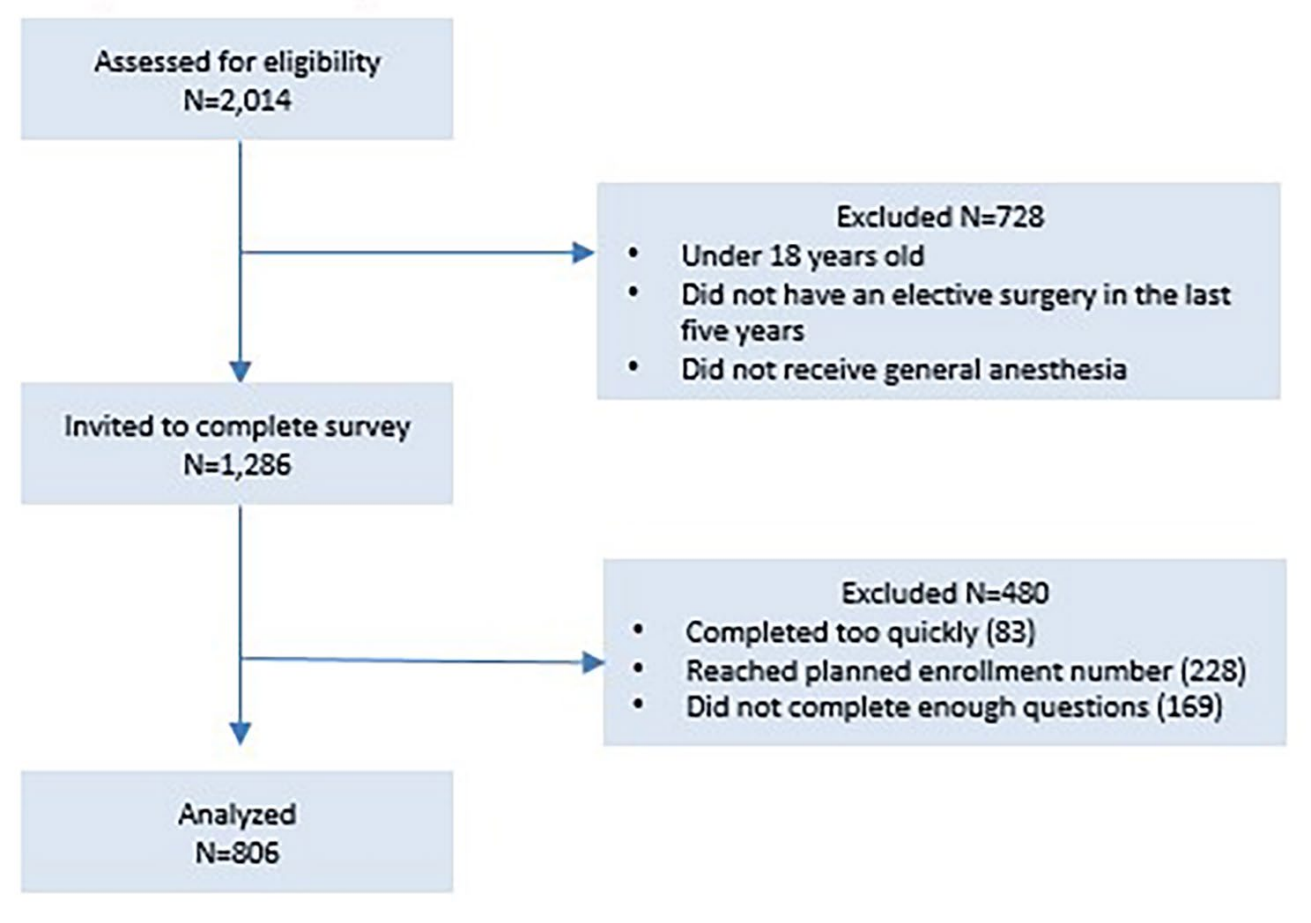
Survey Process

A total of 2,014 respondents entered the survey. Of these, 728 did not meet eligibility criteria and were excluded. Of the remaining 1286 participants, 806 respondents completed the survey (Fig. 1. Survey Process). The majority of respondents were between the ages of 18 and 50 years, and had obtained some college training or a higher degree. Health insurance type and current living community was similar between respondents (Table 1. Demographics).

**Table 1:** Demographics

Demographic Variable	N (%)
**Gender**	
Male Female Non-binary	409 (50.7) 392 (48.6) 5 (0.6)
**Age**	
18–35 years 36–50 years 51–65 years 65 + years	351 (43.6) 275 (34.1) 123 (15.3) 57 (7.1)
**Race and Ethnicity** (select all that apply)	
White or Caucasian Black or African American Hispanic Asian American Indian or Alaska Native Multi-Racial Native Hawaiian or Pacific Islander Other Prefer not to answer	551 (68.4) 141 (17.5) 81 (10.1) 25 (3.1) 15 (1.9) 9 (1.1) 3 (0.4) 61 (7.6) 1 (0.1)
**Education**	
Less than high school Some high school High school diploma or GED Some college or technical training A college degree A graduate or professional degree Prefer not to answer	5 (0.6) 22 (2.7) 218 (27.1) 246 (30.5) 243 (30.2) 71 (8.8) 1 (0.12)
**Health Insurance**	
Medicaid Medicare Private Insurance No Insurance Other Prefer not to answer	243 (30.2) 164 (20.4) 316 (39.2) 58 (7.2) 19 (2.4) 6 (0.7)
**Current Living Community**	
Rural Urban Suburban Prefer not to answer	213 (26.4) 29 (36.2) 300 (37.2) 1 (0.1)

### Attitudes

#### Preferences for anesthesia: important factors

**Fig. 2:**
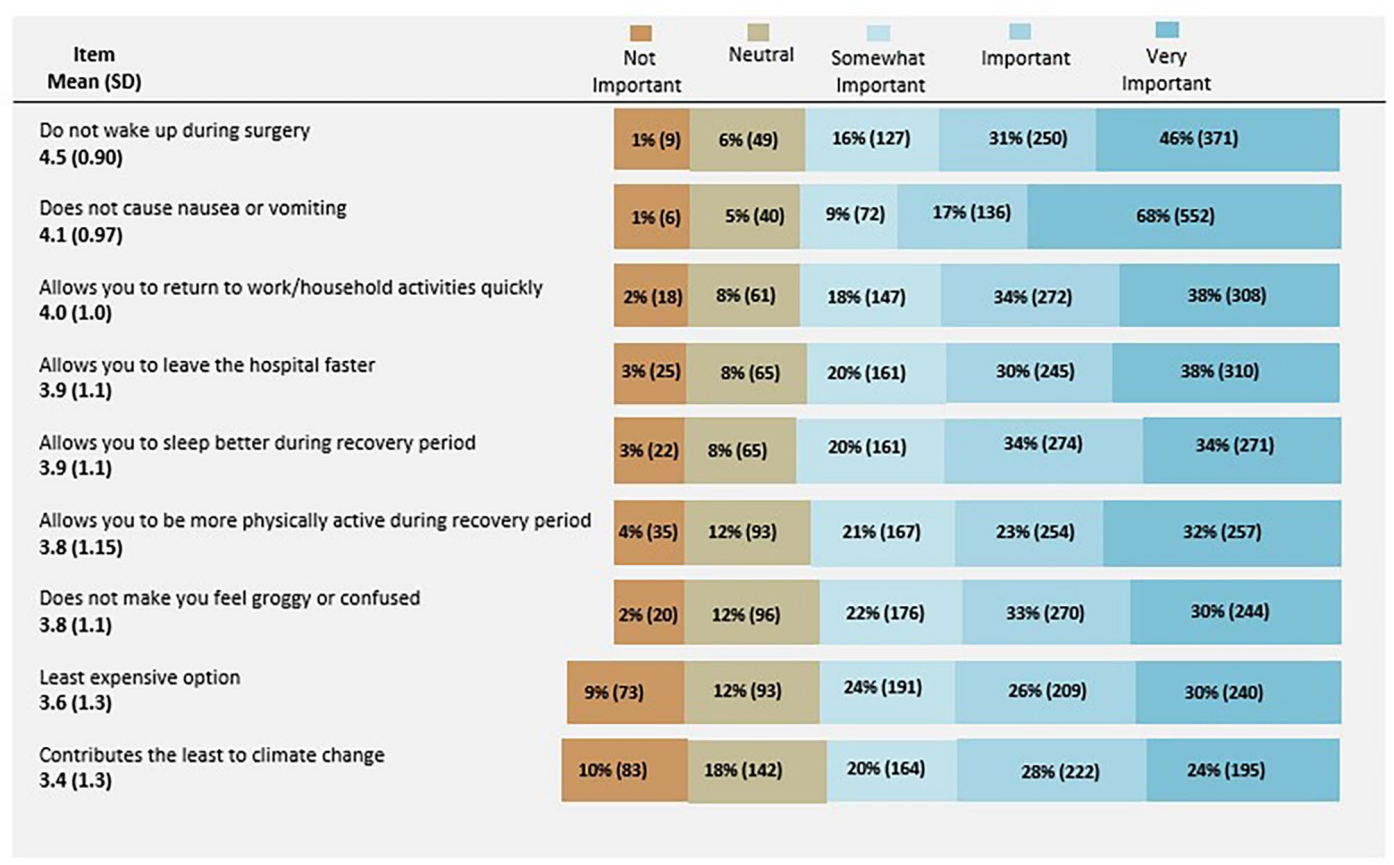
Important Factors When Deciding Between Two Types of Anesthesia N= 806; Determined by respondents on a 5-point Likert scale, where 1 = not important, 2= neutral, 3=somewhat important, 4=important, and 5 = very important

Respondents reported several factors that were important to them when deciding between two types of anesthetics. Ensuring that the type of anesthesia they receive works properly and does not cause them to wake up during surgery and does not cause nausea or vomiting after waking up were categorized as important or very important to 85% and 77% of respondents. These factors also had the highest mean likert scores overall [4.5 (0.90) and 4.1 (0.97), range 1–5]. (Fig. 2. Important Factors when deciding between two different types of anesthetics; important factors were explored based on the Theory of Planned Behavior and past literature; [16–20]).

When comparing by age, respondents > 50 years old had statistically significantly higher mean likert scores for the following importance factors compared to those ≤ 50 years old: do not wake up during surgery and does not make you feel groggy or confused. Conversely, respondents ≤ 50 years old had higher mean likert scores forthe anesthetic contributes least to climate change. (Table 2. Important factors assessed by Age with statistically significant results). Higher mean likert scores were also notable for White respondents in comparison to Black or other races and ethnicities with regards to not waking up and not causing nausea and vomiting. White respondents had the lowest mean likert score for contributions to climate change (Table 3. Important factors assessed by Race with statistically significant results). When comparing these factors by gender, no notable differences were found.

**Table 2:** Important factors assessed by Age with statistically significant results

	Age ≤ 50 years Mean (SD)	Age > 50 years Mean (SD)	P value*
Do not wake up during surgery	4.4 (0.95)	4.6 (0.71)	0.006
Does not cause nausea or vomiting	4.1 (0.99)	4.3 (0.89)	0.078
Does not make you feel groggy or confused	3.7 (1.09)	4.0 (1.03)	0.012
Allows you to leave the hospital faster	3.9 (1.11)	4.1 (1.00)	0.054
Least expensive option	3.6 (1.23)	3.3 (1.40)	0.060
Contributes the least to climate change	3.5 (1.25)	3.0 (1.40)	< 0.001

* Bonferroni corrected *P* values were calculated with the unpaired Student’s t-test

**Table 3:** Important factors assessed by Race with statistically significant results

	White Mean (SD)	Black Mean (SD)	Other Mean (SD)	P value*
Do not wake up during surgery	4.6 (0.76)	4.1 (1.06)	4.2 (1.11)	< 0.001
Does not cause nausea or vomiting	4.2 (0.92)	3.9 (1.12)	4.1 (0.99)	0.018
Contributes the least to climate change	3.2 (1.32)	3.7 (1.19)	3.4 (1.29)	0.024

*Bonferroni corrected *P* value for the F statistic

#### Preferences for anesthesia: concerns

**Fig. 3:**
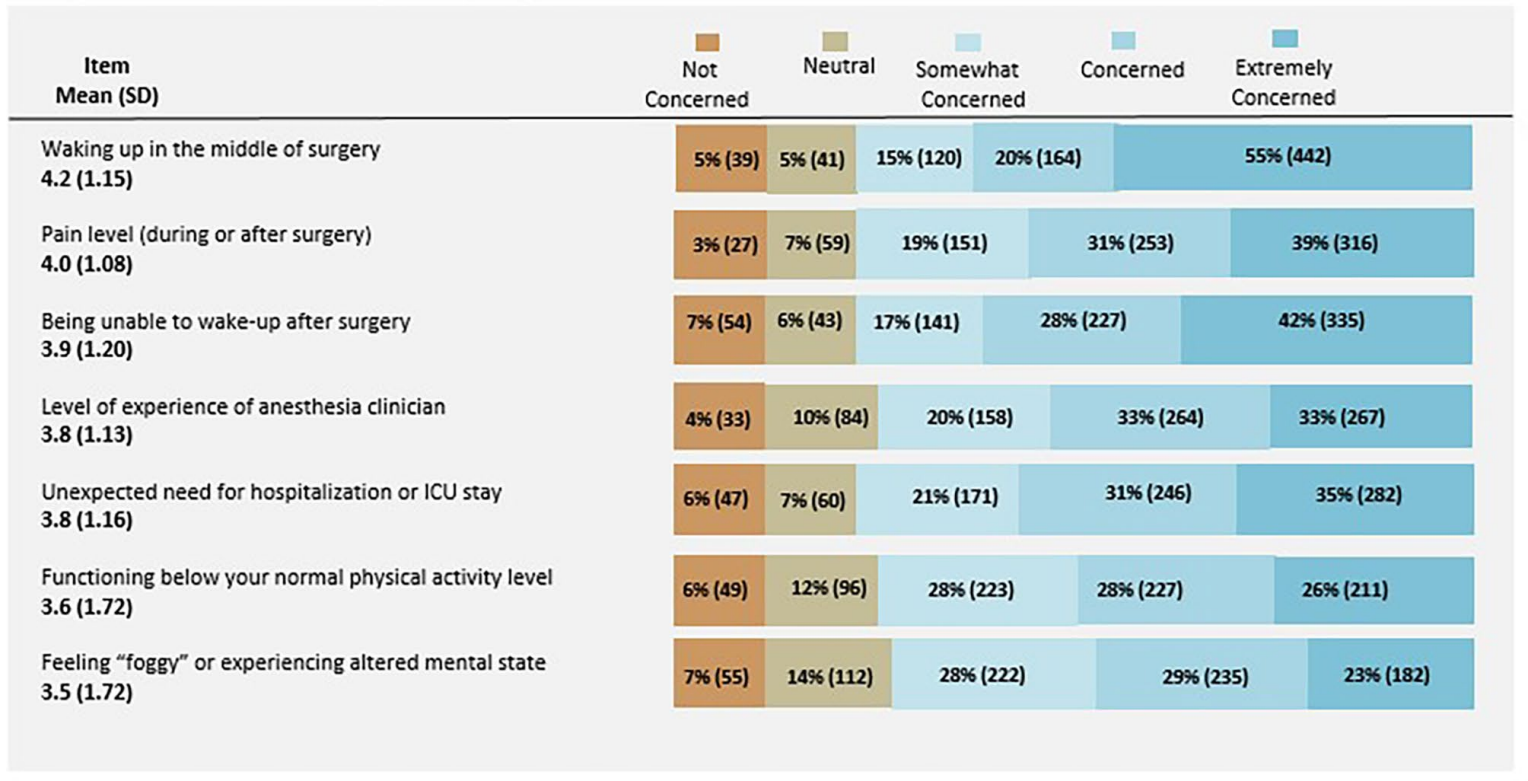
Concerns Regarding Experiences with Anesthesia N= 806; Determined by respondents on a 5-point Likert scale, where 1 = not concerned, 2= neutral, 3=somewhat concerned, 4=concerned, and 5 = extremely concerned

A total of 75% of respondents were concerned or extremely concerned about waking up in the middle of surgery and 70% were concerned or extremely concerned about pain with mean likert scores of 4.2 (1.15) and 4.0 (1.08), range 1–5. (Fig. 3. Concerns Regarding Experiences with General Anesthesia). When comparing concerns by age, gender and race, no differences were found.

#### Preferences for shared-decision making

When asked about the degree of agreement or disagreement regarding the importance of being included in the decision to choose inhaled or intravenous anesthesia during surgery with 1 = strongly disagree to 5 = strongly agree, respondents had an overall mean score of 4 (1.0). No differences by age, gender or race were found.

Based on the control preferences scale, 346 (43%) respondents prefer that the role be patient-led, 225 (28%) prefer shared decision making and 235 (29%) prefer this to be a physician-led process. When assessed by age, more respondents ≤ 50 years of age prefer this process to be patient-led (46%) compared to those > 50 years old (33%, p = 0.0014). Similarly, more Black respondents (55%) prefer a patient-led role compared to White (39%) or other races (45%, p value for the F statistic = 0.006). The preference for shared-decision making did not differ by age or race. No differences between genders were found.

The mean of the responses to the 11 Trust in Physician questions was 72 [12.5, range from 0 (no trust) to 100 (complete trust)]. Respondents who preferred a physician-led role in decision-making had a higher Trust in Physician score of 74.6 (12.8) compared to those that preferred a patient-led role, 70.0 (11.44), p value for the F statistic < 0.0001 suggesting the shared-decision making roles are influenced by trust in physician.

#### Previous surgical and anesthesia experiences & feelings

**Fig. 4:**
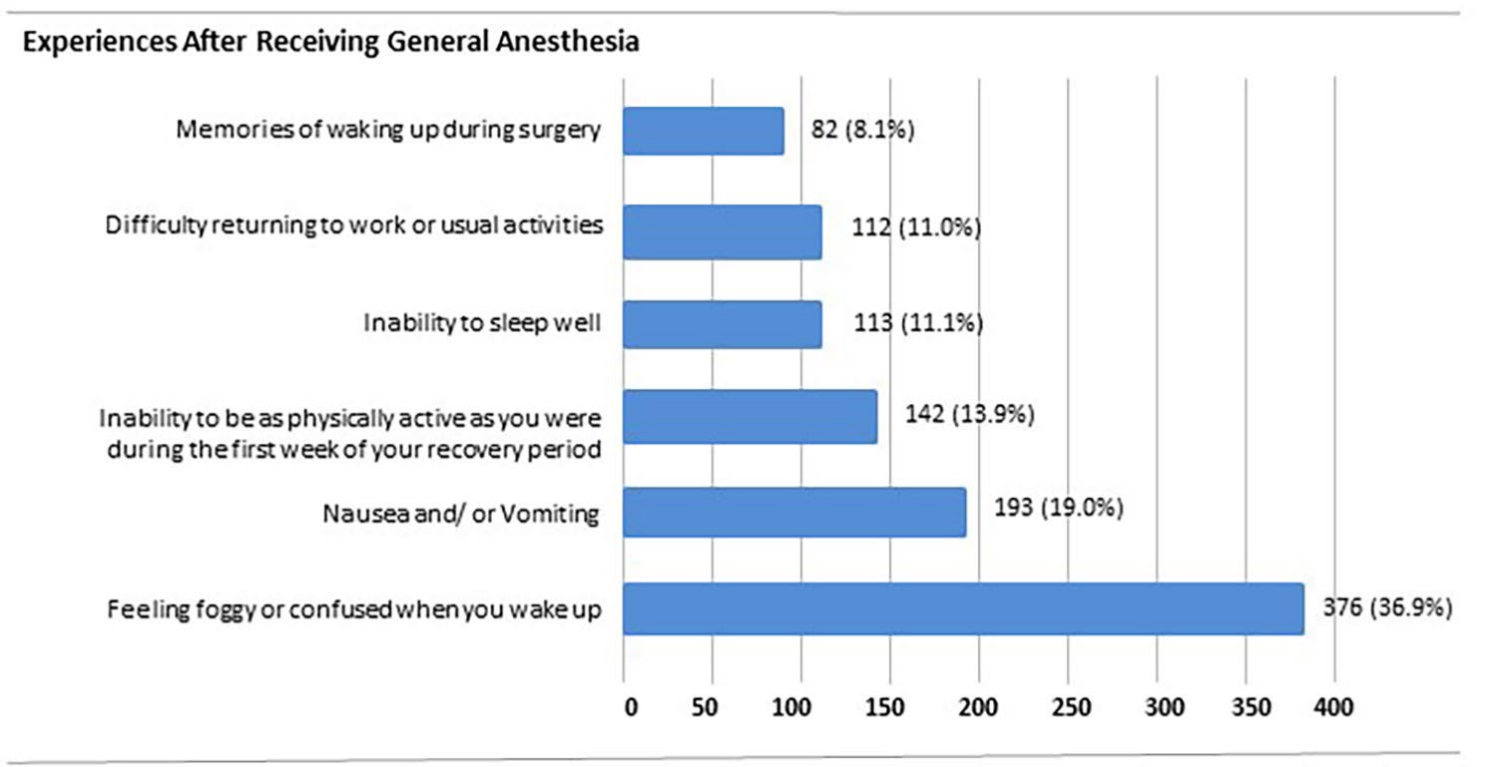
Previous Surgical/Anesthesia Experiences

When asked specifically about negative experiences, respondents reported 1,018 negative experiences after receiving anesthesia. Of these, feeling groggy or confused (37%) and nausea and/or vomiting (19%) were the most common (Fig. 4. Previous Surgical/Anesthesia Experience).

When assessing these experiences by gender, more female patients (119, 30.4%) compared to male patients (73, 17.8%) experienced nausea and vomiting (p < 0.001) and reported feeling groggy or confused when waking up (196, 50% vs. 175, 42.8%; p < 0.007). Interestingly, all experiences except for memories of waking up during surgery were reported more commonly in respondents ≤ 50 years old (See Table 4. Previous Surgical/Anesthesia Experiences assessed by Age). No differences in race were found.

**Table 4:** Previous Surgical/Anesthesia Experiences assessed by Age

	≤ 50 Yrs (n = 626) n (%)	> 50 Yrs (n = 180) n (%)	P Value*
Feeling groggy or confused when you woke up	315 (50.3)	61 (33.9)	< 0.001
Nausea and/or vomiting	169 (27.0)	24 (13.3)	< 0.001
Inability to be as physically active as you were at baseline during the first week of your recovery period	123 (19.6)	19 (10.6)	0.030
Inability to sleep well during the first week of your recovery period	101 (16.1)	12 (6.7)	0.006
Difficulty returning to work or usual home activities	101 (16.1)	11 (6.1)	< 0.001
Memories of waking up during surgery	67 (10.7)	15 (8.3)	1.000

* Bonferroni corrected *P* values were calculated with the unpaired Student’s t-test

The majority of respondents reported feeling satisfied or very satisfied (77.4%) with their recovery experience (3.0 (0.77), range 1–4) and had a good to excellent (83.1%) feeling of well-being after surgery (3.4 (1.0), range 1–5).

A total of 347 (43%) respondents reported feeling moderately to extremely worried about receiving general anesthesia [3.1 (1.3), range 1–5] prior to their procedure. Respondents > 50 years of age had a higher mean worry score than those ≤ 50 years old [3.2 (1.25) versus 2.7 (1.4), p < 0.0001] as did female respondents compared to male [3.2 (1.2) versus 2.9 (1.4), p < 0.0001]. White respondents had a lower mean score of 2.99 (1.3) compared to Blacks [3.3, (1.33)] or other races [3.4 (1.2), p value for F statistic = 0.0013].

### Beliefs

Half of respondents believe that the level of anesthesia clinician experience (54%), dose of anesthesia (53%) and duration of administration (50%) impact whether a patient will have a good experience after receiving general anesthesia.

When respondents were asked questions about general anesthesia, 39.5% believed that both types of anesthesia are safe to administer. They did not think nausea or vomiting, a feeling of general well being, or a quicker time to recovery was associated with one type over another. They believe that intravenous is more commonly given than inhaled. About 40% of respondents thought inhaled anesthesia was associated with a higher incidence of intraoperative awareness and was more likely to contribute to climate change (Table 5. Beliefs about General Anesthesia).

**Table 5:** Beliefs about General Anesthesia

	Intravenous (IV), n (%)	Inhaled gas, n (%)	Both are equal, n (%)	Unsure, n (%)
**Characteristics of Anesthesia Type**				
Safer	220 (27.3)	161 (20.0)	318 (39.5)	107 (13.3)
More Nausea/Vomiting	204 (25.3)	237 (29.4)	206 (25.6)	159 (19.7)
More likely to lead to a feeling of general well-being	221 (27.4)	227 (28.2)	213 (26.4)	145 (18.0)
More likely associated with quicker time to recovery	240 (29.8)	252 (31.3)	174 (21.6)	140 (17.4)
Most commonly given	343 (42.6)	217 (26.9)	174 (21.6)	72 (8.9)
Higher risk of intraoperative awareness	172 (21.3)	306 (38.0)	161 (20.0)	167 (20.7)
More likely to contribute to climate change	100 (12.4)	322 (40.0)	103 (12.8)	281 (34.9)

### Perceived social norms and shared decision making

#### Shared-decision making

When asked if the clinicians who administered anesthesia included them in the decision to choose inhaled versus intravenous anesthesia, a total of 311 (40%) respondents stated they were not included at all [2 (1.1), range 1–4]. When comparing the mean score by age, it was lower in respondents > 50 years old [1.9 (1.1)] compared to those ≤ 50 years old [2.3 (1.1), p = 0.0001]. Similarly, it was lower in White respondents [2.0 (1.1)] compared to Blacks [2.5(1.0)] and other races [2.3 (1.1), p value for the F statistic < 0.0001]. No differences between gender were found.

When assessing shared decision making behaviors, a total of 7.8% participants reported the highest score across all three collabo-RATE items indicating that shared decision making about anesthetic options rarely occurred. Despite these low scores, a total of 590 (73.2%) respondents stated that their anesthesia clinicians encouraged them to ask questions and 671 (83.3%) stated the anesthesia clinician made them feel relaxed. More respondents ≤ 50 years old stated anesthesia clinicians encouraged them to ask questions compared to those > 50 years of age (75% versus 67%, p = 0.03). No differences in gender or race were found.

When respondents were asked whether or not they agree that most surgical patients want to know more about anesthesia options, 697 (86%) agreed or strongly agreed [mean score 3.17 (0.71), range 1–4]. Similarly, 700 (86%) agreed or strongly agreed that most surgical patients would feel comfortable engaging in the decision process to select inhaled or intravenous anesthesia [mean score 3.19 (0.71), range 1–4]

### Self-efficacy

#### Confidence in decision-making

Using a modified version of the decision self-efficacy scale, respondents had an overall mean score of 76.6 (15.9). When comparing this score by gender, age and race, no differences were found.

#### Confidence in information seeking

When respondents were asked if they had ever looked for information about general anesthesia, 384 (47.6%) stated yes. Of these 384 respondents, there were a total of 585 places they searched; online websites (412, 70%) were most commonly sought. Seeking in person or telephone discussions with a healthcare team member (52, 9%) and printed pamphlets (3, 0.5%) were rare. In addition, 248 (65%) of these patients agreed that it took a lot of effort to get the information they needed [2.2 (0.98), range 1–4] and 205 (53%) agreed to feeling frustrated during the search [2.4 (0.99), range 1–4].

When stratified by age, significantly more respondents ≤ 50 years old searched for information compared to those > 50 years (394, 56% versus 35, 19.4%; p < 0.001). Patients > 50 years also had higher mean scores for effort amount [2.6 (0.91) vs. 2.1 (0.98), p value = 0.005] and feelings of frustrations [2.8 (0.98) vs. 2.4 (0.99), p value = 0.04] during their search than those < 50 years old. No differences were found when stratified by race or gender.

## Discussion

Although there are some known advantages and disadvantages of propofol TIVA and inhaled volatile anesthesia [38–42], there are gaps in the evidence about the recovery experiences and safety-related aspects of the general anesthetic techniques. In most cases, there is not a clear superior option from a clinical perspective, outside of known, rare contraindications such as allergies to propofol or malignant hyperthermia and severe postoperative nausea and vomiting with inhaled volatile agents. Thus, both clinician and patient preferences are central to the decision. Shared decision making involves engaging patients with evidence-based information, eliciting preferences (including preferences for involvement in the choice), and deliberating on a decision together, before making a final choice based on the evidence and patients’ goals and preferences [43].

In this cohort of 806 patients who received general anesthesia for an elective procedure in the last five years, respondents indicated that they want to be engaged in the decision of which anesthetic agent they receive. More respondents in this study prefer for this decision to be patient-led than physician-led or a shared responsibility, which is consistent with previous studies assessing patient preferences for decision making [43–46]. Respondents that preferred a patient-led role had lower mean Trust in Physician scores suggesting that trust in anesthesia clinicians may influenced shared-decision making role preferences. Although anesthesia clinicians made respondents feel relaxed and encouraged them to ask questions, most respondents were not included in the decision to choose inhaled versus intravenous anesthesia for their procedure. Older respondents (> 50 years) and White respondents were less likely than younger adults and other races to feel included highlighting the possibility of age and racial disparities occurring during the decision making process.

Only 7.8% reported engaging in full shared decision making about anesthesia. Respondents were confident in their ability to obtain information and discuss choices and concerns with clinicians, likely influencing their preference to be engaged in the decision making process. However, obtaining this information was not always easy. The most commonly searched source for information about general anesthesia in our study was the internet and the process took a lot of effort, leading to frustration. This finding highlights the need for clear and easily accessible patient facing online educational materials that can facilitate a patient’s ability to engage in the anesthesia decision process. More respondents ≤ 50 years old compared to those > 50 years old searched online for information in our study highlighting that information search strategies may differ by age. Similarly, a previous survey noted that only 28% of adults ages 50–80 years old who were considering elective surgery used the internet to find information about their surgery [47]. Additionally, older patients in our study felt it took more effort to find information they needed and felt frustrated during the process when compared to younger patients. In order to meet the needs of patients of all ages seeking general anesthesia information from varying sources, it is important that patient-facing educational material be created and accessible.

Several preference factors pertaining to general anesthetic types were identified. Intraoperative awareness, pain, nausea, vomiting and quickly returning to work and usual household activities were important to respondents. These findings are similar to previous studies assessing patients’ anesthesia related fears [9, 48–50, 47]. In addition, age and race may also influence factors important to patients. In our study, older respondents (> 50 years) were more concerned than younger respondents about intraoperative awareness, nausea or vomiting, or feelings of confusion or grogginess. They also emphasized the importance of leaving the hospital quickly, compared to those < 50 years old. Conversely, cost and the impact on the environment were factors more important to those < 50 years old. These differences could help anesthesia clinicians consider specific patient groups and preferences when discussing anesthetic choice.

When reflecting on their previous anesthesia experiences, respondents in this study reported an overall positive experience and were satisfied with their recovery experience. Notably, respondents > 50 years old, females and those of races other than White or Black worried more about receiving general anesthesia compared to younger respondents, males, White or Black respondents. These groups may require further reassurance or preoperative discussions aimed at understanding and alleviating reasons for concern.

Overall, most respondents reported that after receiving general anesthesia, they felt groggy or confused and experienced nausea and/or vomiting. More female patients compared to male patients experienced nausea and vomiting and reported feeling groggy compared to males. This is consistent with previous literature suggesting females have a higher risk for postoperative nausea and vomiting and poorer quality of recovery [30–32, 51]. Interestingly, all experiences except for memories of waking up during surgery were more commonly reported in patients ≤ 50 years compared to those > 50 years. Although this may seem contradictory to what one might assume, it is important to note that we did not take into consideration the type of surgery and other patient factors that could influence patient experiences in this survey.

Although feeling groggy or confused when waking up and experiencing nausea and/or vomiting was common overall, these factors were not the most commonly chosen importance factors. Instead, it was important that the type of anesthesia received does not cause them to wake up during surgery. Similarly, waking up in the middle of surgery was the most commonly reported concern, despite this experience being reported 8% of the time. Reports of possible and definite intraoperative awareness incidence ranges from 0.001 to 0.32% in retrospective studies, meta analyses and randomized controlled trials with general anesthesia [52–61]. Although this complication is rare, it is still very concerning to patients. In this cohort of surgical patients, previous experiences did not necessarily drive their preferences. It remains unclear whether TIVA or inhaled anesthesia is safer with regard to intraoperative awareness incidence, despite 40% of respondents in this study believing it to be more common with inhaled anesthesia. Previous reports, aimed at clinicians, highlight the importance of ensuring appropriate administration of anesthesia and receiving adequate education pertaining to administration techniques in order to prevent important safety concerns such as intraoperative awareness [2, 52–61]. Of note, patients in our study also recognize that clinician controlled factors including anesthetic dose given, anesthetic duration, and clinician experience may impact a patient’s experience.

The findings of our study should be interpreted in the context of a few study limitations. When comparing our study with the results of previous studies [9, 48–50] it is important to note that methodological aspects differ, including specific attributes and answer choices examined, instruments used to examine questions, and varying statistical analyses and presentations of results. In addition, although there was a statistically significant difference in the relationship between trust and preferences for shared decision-making, trust was reasonably high for both those who preferred and did not prefer high engagement in decisions. However, when we reviewed other studies on trust in physicians, many describe small differences as meaningful because most patients will not want to say they do not trust a physician at all, having placed care in their hands [25]. Mean trust in general ranges from about 45 (lower end of trust) to 83 (higher end of trust), consistent with our study. Moreover, given the relatively high number of those reporting awareness during surgery, it is possible that some had regional or sedation anesthesia rather than general anesthesia [62]. It is also possible that patients reported awareness based on memories that occurred around the time of surgery, such as after waking up in the operating or recovery room, but not during the surgery itself. Previous studies emphasize the importance of further questioning and expert review to determine whether or not definite intraoperative awareness with recall has occurred [53, 63]. Future work should explore experiences of awareness during surgery in more detail. Despite these acknowledged limitations, intraoperative awareness, not waking up, nausea and vomiting, and pain are still common concerns experienced by patients undergoing surgery with general anesthesia.

## Conclusions

For those that were involved in the decision, it is unclear if they were able to make an informed choice since trade-offs between each option have not been fully elucidated. This emphasizes an important call to action and immediate necessity to study patient experiences in a rigorous manner in order to take the first steps in being able to provide patients with the information they need to make a choice. This could initiate the process of narrowing the gap between patient and anesthesia clinician shared decision making. The upcoming THRIVE trial will explore which type of anesthesia (Propofol TIVA or inhaled volatile anesthesia) results in a better patient experience (NCT05346588). If one type of anesthesia was shown to be superior with regards to recovery experiences and intraoperative awareness, mitigation of common patient fears could occur. In addition, the information from this trial can inform future shared decision-making tools, informed consent, and educational materials and framing of anesthetic choices for patients. Key outcomes are important to stakeholders in the informed consent process including knowledge, decision-making, communication, trust, and process [64]. Such a transformative approach to care can immediately impact the care experience for millions of people worldwide.

### Electronic supplementary material

Below is the link to the electronic supplementary material.

Supplementary Material 1

## Data Availability

The datasets generated during and/or analyzed during the current study are available from the corresponding author on reasonable request.

## Abbreviations

THRIVE: Trajectories of Recovery after Intravenous Propofol vs. inhaled VolatilE anesthesia
TIVA: Total intravenous anesthesia
