# Supplementary material for: A survey of surgical patients’ perspectives and preferences towards general anesthesia techniques and shared-decision making
Source: BMC Anesthesiol. 2023 Aug 17;23:277. doi: 10.1186/s12871-023-02219-5 (PMC10433576; doi:10.1186/s12871-023-02219-5)
Supplement: Supplementary file 1 — Supplementary Material 1 [file 12871_2023_2219_MOESM1_ESM.docx]

**Appendix 1. Survey Questions**

***Screening questions***

- Are you 18 years of age or older? (*Y/N)*
- Have you ever had either IV or inhaled anesthesia for an elective surgery in the last year? (*Y/N)*

***Preferences:***

- If you were deciding between two different types of anesthesia, how important is it that the following is true? *Scale of 1-5 (not at all important to very important). The type of anesthesia:*
  - works properly and you do not wake up during surgery
  - does not make you feel groggy or confused when you wake up
  - does not make you nauseous or vomit after you wake up
  - allows you to return to work or usual household activities quickly
  - is the least expensive option
  - allows you to leave the hospital faster
  - allows you to sleep better during the first week of your recovery period
  - allows you to be more physically active during the first week of your recovery period
  - contributes the least to climate change and global warming
- How concerning are the following to you regarding your experience with general anesthesia? Please rate each item from *“not concerned at all” to “extremely concerned”*
  - Being unable to wake-up after surgery
  - Level of experience of the clinician administering anesthesia
  - Unexpected need for hospitalization or ICU stay
  - Pain level (during or after surgery)
  - Waking up in the middle of surgery
  - Feeling “foggy” or experiencing altered mental state
  - Functioning below your normal physical activity level after surgery

***Previous Surgical Experience:***

Thinking back to when you had surgery,

- How worried were you about receiving general anesthesia? *(not at all, slightly, somewhat, moderately, extremely)*
- Did you experience any of the following after receiving anesthesia? (check all that apply)
  - Memories of waking up during surgery
  - Feeling groggy or confused when you woke up
  - Nausea and/or vomiting
  - Difficulty returning to work or usual home activities
  - Inability to sleep well during the first week of your recovery period
  - Inability to be as physically active as you were at baseline during the first week of your recovery period
- How satisfied were you with how quickly you were back to your baseline level of activities? *(Not at all satisfied, a little satisfied, satisfied , very satisfied)*
- How would you rate your overall feeling of well-being after surgery? *(Excellent, Very good, Good, Fair, Poor)*

***Previous Anesthesia Clinician Experience***

- Did the clinician who administered anesthesia encourage you to ask questions? *(Yes, no, unsure)*
- Did talking with the clinician who administered anesthesia make you feel more calm and relaxed? *(Yes, no, unsure)*
- How much, if at all, did the clinician who administered anesthesia include you in the decision to choose inhaled versus IV anesthesia? *(not at all, a little, somewhat, a lot)*
- How much effort was made to help you understand the differences between inhaled or intravenous/IV anesthesia? *(no effort was made, a little effort was made, some effort was made, a lot of effort was made, every effort was made)*
- How much effort was made to listen to the things that matter most to you about receiving anesthesia?*(no effort was made, a little effort was made, some effort was made, a lot of effort was made, every effort was made)*
- How much effort was made to include what matters most to you in choosing which type of anesthesia (inhaled or IV) to receive? *(no effort was made, a little effort was made, some effort was made, a lot of effort was made, every effort was made)*

***Shared Decision Making:***

- How much do you agree or disagree with this statement “It is important to me that I am included in the decision about which type of anesthesia (inhaled or intravenous/IV) I receive during surgery”? *(Strongly agree, somewhat agree, neither agree nor disagree, somewhat disagree, strongly disagree)*
- What role do you prefer to take in deciding which type of anesthesia you will receive for surgery? Mark only one:
  1. I prefer to make the decision with little or no input from the clinician administering anesthesia.
  2. I prefer to make the decision after seriously considering the opinion of the clinician administering anesthesia.
  3. I prefer that the clinician administering anesthesia and I share responsibility for the decision together.
  4. I prefer the clinician administering anesthesia to make the decision after seriously considering my opinion.
  5. I prefer to leave all decisions about my treatment to the clinician administering anesthesia.

***Beliefs & Perceived Social Norms***

- How much do you believe that each of the following can influence whether or not a patient will have a *good* experience (minimal to no side effects and a quick time to full recovery) after receiving intravenous/IV anesthesia?
  1. Level of experience of the clinician administering anesthesia *(a lot, a little, not at all, don’t know)*
  2. The dose of the anesthesia being given *(a lot, a little, not at all, don’t know)*
  3. The duration of the time the anesthesia is given *(a lot, a little, not at all, don’t know)*
- I believe most surgical patients want to know more about the differences between anesthesia options. *(Strongly disagree, disagree, agree, strongly agree)*
- I believe most surgical patients would feel comfortable engaging in the decision process selecting inhaled or intravenous/IV anesthesia. *(Strongly disagree, disagree, agree, strongly agree)*

***Self-efficacy:***

Please rate your confidence (scale 1-4) in the following statements:

- Regarding inhaled or intravenous/IV anesthesia, I feel confident that I can: *(not at all confident, somewhat confident, confident, very confident)*
  - Get the facts about the benefits of each choice of anesthesia (inhaled or intravenous/IV)
  - Get the facts about risks and side effects of each choice of anesthesia (inhaled or intravenous/IV)
  - Understand the information enough to be able to make a choice
  - Ask questions without feeling dumb
  - Express my concerns about each choice
  - Let the clinic team know what’s best for me.

***Information Seeking about Anesthesia:***

We would like to know about your experience in searching for information about anesthesia.

- Have you ever looked for information about general anesthesia (inhaled or intravenous/IV) from any source? (Y/N)

1. If Y → Where did you look for information? Check all that apply:

- Online website with text
- Online video
- In-person or telephone discussion with a member of your health care team
- Printed pamphlet
- Another place
  - Please tell us where: _______________

1. Based on the results of your most recent search for information about general anesthesia, how much do you agree or disagree with each of the following statements?
   1. It took a lot of effort to get the information you needed

(strongly agree, somewhat agree, somewhat disagree, strongly disagree)

- 1. You felt frustrated during your search for information

(strongly agree, somewhat agree, somewhat disagree, strongly disagree)

1. If no → Overall, how confident are you that you could get advice or information about general anesthesia if you needed it?

(Completely Confident, Very Confident, Somewhat Confident, A little Confident, Not confident at all)

**Perceptions about Anesthesia:**

- Which type of anesthesia is safer to administer to patients? *(IV (Intravenous), Inhaled Gas, Both are equal, Unsure)*
- Which type of anesthesia is most commonly given by clinicians? *(IV (Intravenous), Inhaled Gas, Both are equal, Unsure)*
- Which type of anesthesia is more likely to contribute to climate change? *(IV (Intravenous), Inhaled Gas, Both are equal, Unsure)*
- Which type of anesthesia is more likely to cause nausea or vomiting? *(IV (Intravenous), Inhaled Gas, Both are equal, Unsure)*
- Which type of anesthesia is more likely to lead to a feeling of general well-being after a procedure?*(IV (Intravenous), Inhaled Gas, Both are equal, Unsure)*
- Which type of anesthesia is more likely to be associated with a quicker time to recovery after a procedure? *(IV (Intravenous), Inhaled Gas, Both are equal, Unsure)*

Intraoperative awareness refers to waking up during surgery. If this happens, a patient can recall their surroundings, or an event related to the surgery, while under general anesthesia.

- Which type of anesthesia has a higher risk of experiencing awareness during surgery? *(Intravenous), Inhaled Gas, Both are equal, Unsure)*

***Medical Mistrust Questions:***

Please review these statements below and indicate your agreement/disagreement regarding your perceptions about clinicians in general.

- - - 1. I doubt that my clinician really care about me as a person *(Strongly disagree, disagree, neutral, agree, strongly agree)*
      2. My clinician is usually considerate of my needs and puts them first *(Strongly disagree, disagree, neutral, agree, strongly agree)*
      3. I trust my clinician so much I always try to follow his/her advice *(Strongly disagree, disagree, neutral, agree, strongly agree)*
      4. If my clinician tells me something is so, then it must be true *(Strongly disagree, disagree, neutral, agree, strongly agree)*
      5. I sometimes distrust my clinician’s opinions and would like a second one *(Strongly disagree, disagree, neutral, agree, strongly agree)*
      6. I trust my clinician’s judgements about my medical care *(Strongly disagree, disagree, neutral, agree, strongly agree)*
      7. I feel my clinician does not do everything he/she should about my medical care *(Strongly disagree, disagree, neutral, agree, strongly agree)*
      8. I trust my clinician to put my medical needs above all other considerations when treating my medical problems *(Strongly disagree, disagree, neutral, agree, strongly agree)*
      9. My clinician is well qualified to manage (diagnose and treat or make an appropriate referral) medical problems like mine *(Strongly disagree, disagree, neutral, agree, strongly agree)*
      10. I trust my clinician to tell me if a mistake was made about my treatment *(Strongly disagree, disagree, neutral, agree, strongly agree)*
      11. I sometimes worry that my clinician may not keep the information we discuss totally private. *(Strongly disagree, disagree, neutral, agree, strongly agree)*
